# A probable Keplerian disk feeding an optically revealed massive young star

**DOI:** 10.1038/s41586-023-06790-2

**Published:** 2023-11-29

**Authors:** Anna F. McLeod, Pamela D. Klaassen, Megan Reiter, Jonathan Henshaw, Rolf Kuiper, Adam Ginsburg

**Affiliations:** 1 Centre for Extragalactic Astronomy, Department of Physics, Durham University, Durham, UK; 2 Institute for Computational Cosmology, Department of Physics, University of Durham, Durham, UK; 3 UK Astronomy Technology Centre, Royal Observatory, Edinburgh, Edinburgh, UK; 4 Department of Physics and Astronomy, Rice University, Houston, TX USA; 5 Astrophysics Research Institute, Liverpool John Moores University, Liverpool, UK; 6 Max Planck Institute for Astronomy, Heidelberg, Germany; 7 Faculty of Physics, University of Duisburg-Essen, Duisburg, Germany; 8 Department of Astronomy, University of Florida, Gainesville, FL USA

**Keywords:** Astrophysical disks, Stars

## Abstract

The canonical picture of star formation involves disk-mediated accretion, with Keplerian accretion disks and associated bipolar jets primarily observed in nearby, low-mass young stellar objects (YSOs). Recently, rotating gaseous structures and Keplerian disks have been detected around several massive (*M* > 8 *M*_⊙_) YSOs (MYSOs)^1–4^, including several disk-jet systems^5–7^. All the known MYSO systems are in the Milky Way, and all are embedded in their natal material. Here we report the detection of a rotating gaseous structure around an extragalactic MYSO in the Large Magellanic Cloud. The gas motion indicates that there is a radial flow of material falling from larger scales onto a central disk-like structure. The latter exhibits signs of Keplerian rotation, so that there is a rotating toroid feeding an accretion disk and thus the growth of the central star. The system is in almost all aspects comparable to Milky Way high-mass YSOs accreting gas from a Keplerian disk. The key difference between this source and its Galactic counterparts is that it is optically revealed rather than being deeply embedded in its natal material as is expected of such a massive young star. We suggest that this is the consequence of the star having formed in a low-metallicity and low-dust content environment. Thus, these results provide important constraints for models of the formation and evolution of massive stars and their circumstellar disks.

## Abstract (web summary)

The authors suggest that a probable Keplerian disk is feeding an optically revealed massive young stellar object in the Large Magellanic Cloud.

## Main

The lack of observations of optically revealed massive (*M* > 8 *M*_⊙_) young stellar objects (MYSOs) is a consequence of the rapid timescales on which massive stars form: they form in heavily embedded regions full of gas and dust, such that the accretion phase typically occurs before the star has time to become exposed due to stellar feedback, whether internal or external. The primary reason for the lack of observations of extragalactic accretion disks around forming stars has been the limited spatial resolution of both ground- and space-based observatories.

At a distance of 50 kpc, the Large Magellanic Cloud (LMC) is a convenient environment for searching for the extragalactic counterparts of the accreting MYSOs known in the Milky Way. For example, there is the recent detection of subparsec scale molecular outflows^8,9^, as well as the discovery of HH 1177, a collimated bipolar jet driven by an MYSO^10^. Although the detection of a molecular outflow from a forming star does not necessarily imply the presence of an accretion disk, collimated jets are generally taken as clear signposts for ongoing disk accretion. To date, there has been no direct detection of a rotating circumstellar Keplerian (accretion) disk or toroid in an external galaxy, making the HH 1177 star/jet system an ideal target to search for these. The Atacama Large Millimeter Array (ALMA) now enables the high-sensitivity and high-angular-resolution observations needed to detect and resolve rotating circumstellar gas in extragalactic MYSOs.

**Fig. 1:**
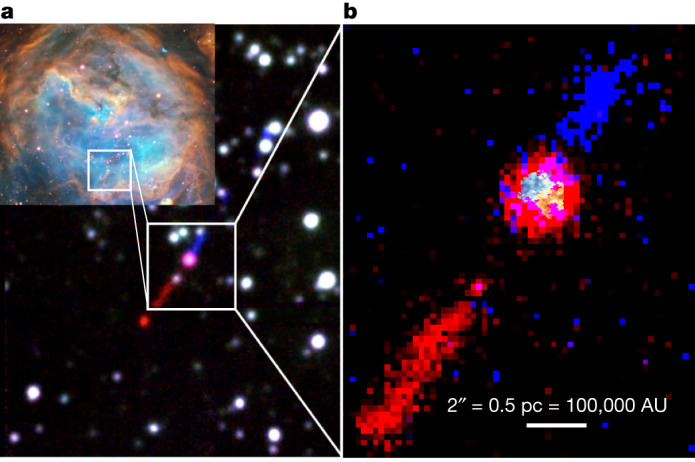
RGB composites of the star-forming region N180 and the jet. **a**, Inset, three-colour composite of the star-forming region N180 in the LMC (red, [S ii] 6,717; green, Hα; blue, [O iii] 5,007), as observed with the MUSE instrument on the Very Large Telescope^10^. **a**, Redshifted and blueshifted wings of the Hα emission line highlighting the externally irradiated HH 1177 jet emerging from the central star. **b**, Same as the main part of **a** but continuum-subtracted and zoomed in onto the driving star of the HH 1177 system, with the CS velocity map overlaid to show the rotating molecular gas at the location of the central star (note that, for illustrative purposes, the CS velocity map has been enlarged by a factor of approximately 4).

**Fig. 2:**
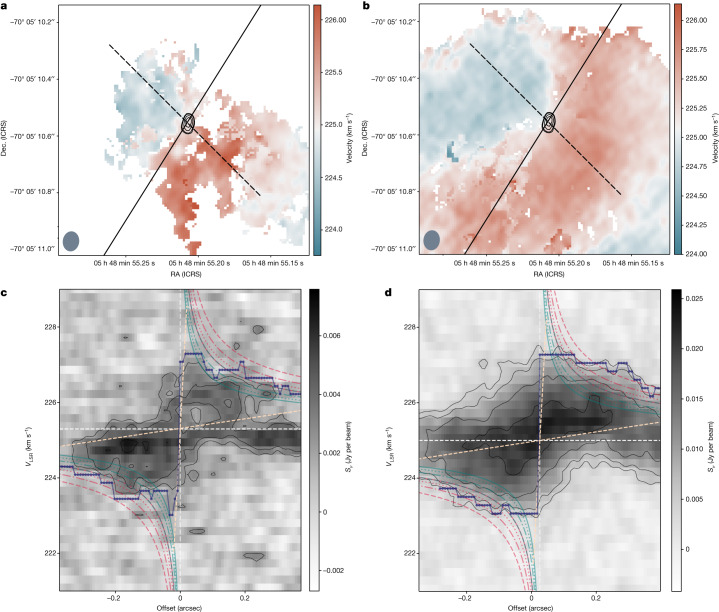
Kinematics of the molecular gas. **a**,**b**, Velocity maps of CS (**a**) and ^13^CO (**b**) derived from SCOUSEPY and ACORNS decompositions (Methods). The velocity corresponds to the local standard of the rest velocity *v*_LSR_. Black contours indicate the location of the continuum source and are shown at 0.17, 0.18, 0.21, 0.25 and 0.28 mJy per beam. The black dashed line corresponds to the 0.75″ and 55° position-angle slit used to extract the PV diagram. It is approximately perpendicular to the optical jet of the HH 1177 system, indicated by the solid black line^10^. **c**,**d**, PV diagrams extracted along the slits shown in **a** and **b** for CS (**c**) and ^13^CO (**d**) (shown is the surface brightness, *S*_ν_). The dashed white lines correspond to the adopted central velocity and position of the source (*v*_LSR_ = 225.3 km s^−1^, 05 h 48 min 55.2099 s − 70 h 05 min 10.579 s). The dashed salmon line indicates the rotating structure’s assumed inner and outer limits, 1,000 and 25,000 AU, respectively. The dark blue points and line indicate the outer envelope (the outer edge^5,14^). The sequence of teal and pink lines shows, respectively, the Keplerian $(v\propto \sqrt{M/R})$ and free-fall $(v\propto \sqrt{2M/R})$ velocity curves for a central source of 10, 12, 15 and 20 *M*_⊙_ (solid, dotted, dot-dashed and dashed, respectively). The series of displayed central star masses encompasses a slightly less massive option to the value of 12 *M*_⊙_ inferred from Spitzer data^13^, as well as masses of the order of what is derived in this work (Methods). Dec., declination; ICRS, International Celestial Reference System; RA, right ascension.

The rotating structure reported here is feeding the central star of the HH 1177 system, which was previously detected in optical integral field spectroscopic observations^10^. The system is in the LMC star-forming region N180, a classical H ii region photoionized by the OB association LH 117 (refs. ^11,12^). HH 1177 has a bipolar (externally) ionized jet with a total (projected) extent of 11 pc originating from a central source (Fig. 1). It is classified as an MYSO with a mass of 12 *M*_⊙_, as estimated from fitting of the infrared spectral energy distribution (SED)^13^. These results indicate that the central star is probably a B-type rather than an O-type star due its current evolutionary stage. The central star of HH 1177 has formed at the tip of a pillar-like molecular cloud structure, oriented towards (in projection) three massive stars of the LH 117 star cluster. HH 1177 remains the only known extragalactic MYSO/jet system and is a unique laboratory for studying MYSO formation and evolution in an external galaxy. In 2019 and 2021, the central star of HH 1177 was targeted with ALMA Band 7 (275–373 GHz) in two different configurations, which captured emission on different size scales. Combined, these observations resulted in a continuum angular resolution of 50 mas × 40 mas (2,500 AU at the distance of the LMC). The ALMA observations covered the molecular lines ^12^CO (*J* = 3 → 2), ^13^CO (*J* = 3 → 2), CS (*J* = 7 → 6) and CH_3_CN (*J* = 18 → 17). In addition to the molecular lines, a fourth spectral window centred on 0.870 mm was devoted to continuum observations, which found a compact, marginally resolved, continuum source at the location of the central star of HH 1177. The continuum source (shown as contours in Fig. 2a,b) has a peak flux of 0.28 mJy per beam, an integrated flux of 0.34 mJy, and a deconvolved size of 50 mas × 32 mas at a position angle of 60°.

We did not detect CH_3_CN emission, and there are no spurious line detections of other species within the spectral windows. Velocity maps of CS and ^13^CO (Fig. 2a,b) were obtained from a multi-component spectral decomposition that was necessary due to the presence of several velocity components, which complicated the analyses (Methods). The velocity maps trace the dense gas kinematics and show a distinct velocity gradient almost perpendicular to the red and blue lobes of the ionized jet. The jet orientation is indicated by the solid black lines in Fig. 2a,b. The red and blue jet lobes have position angles of 144° and −32° with respect to the central source, respectively. We used a position angle of 55°, that is, approximately 90° with respect to the jet, to extract position–velocity (PV) diagrams for both lines.

**Extended Data Fig. 1:**
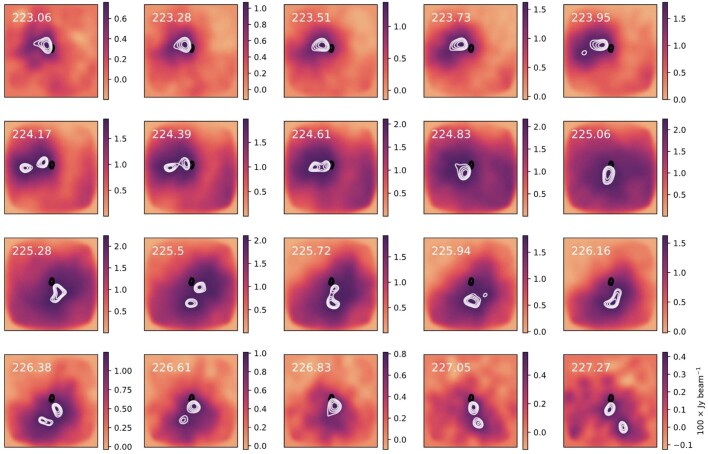
Channel maps of the ^13^CO data cube. Velocities are indicated in each panel. Black contours show the continuum as in Fig. 2; white contours trace the intensity of the colourmaps (for simplicity, only ten contours of the top three percentile intensity values in each channel are shown).

**Extended Data Fig. 2:**
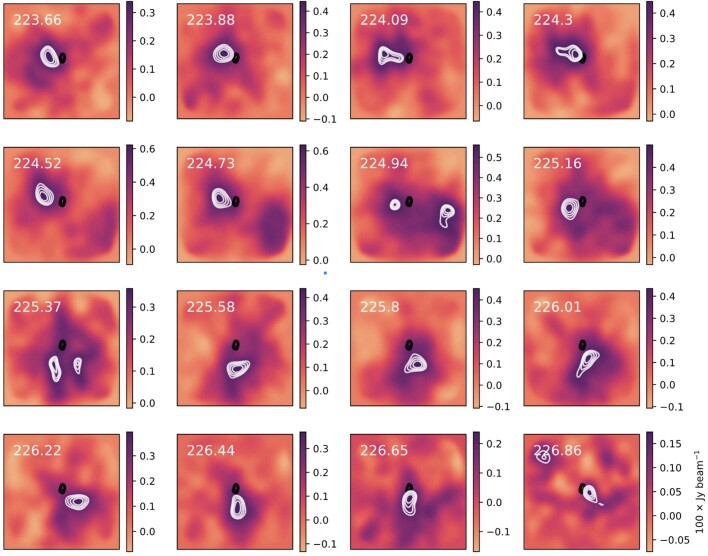
Channel maps of the CS data cube. Velocities are indicated in white in each panel. Black contours show the continuum as in Fig. 2; white contours trace the intensity of the colourmaps (for simplicity, only ten contours of the top three percentile intensity values in each channel are shown).

The PV diagrams (Fig. 2c,d) show the characteristic ‘butterfly’ shape typical of a rotating structure, with higher gas velocities closer to the centre, which is consistent with velocities *v*_rot_ ∝ *R*^*−α*^, with *α* > 0. For both CS and ^13^CO, the kinematics along the outer edge of the emission (dotted dark blue line in Fig. 2c,d; Methods) exhibits Keplerian motion (*α* = 0.5) in the innermost regions (offsets of less than 0.12 arcsec or 0.029 pc or approximately 6,000 AU). The kinematics are consistent with pure free fall (*α* = 1) in the outer parts of the structure. Further insight into the gas kinematics is gained from the channel maps (Extended Data Figs. 1 and 2). With systemic velocities of 225.3 and 225.2 km s^−1^ derived for CS and ^13^CO, respectively, the channel maps confirm that the highest velocity gas is near the central star. The kinematics are, therefore, indicative of the molecular lines tracing a rotating gaseous structure around the MYSO launching the jet of HH 1177.

**Fig. 3:**
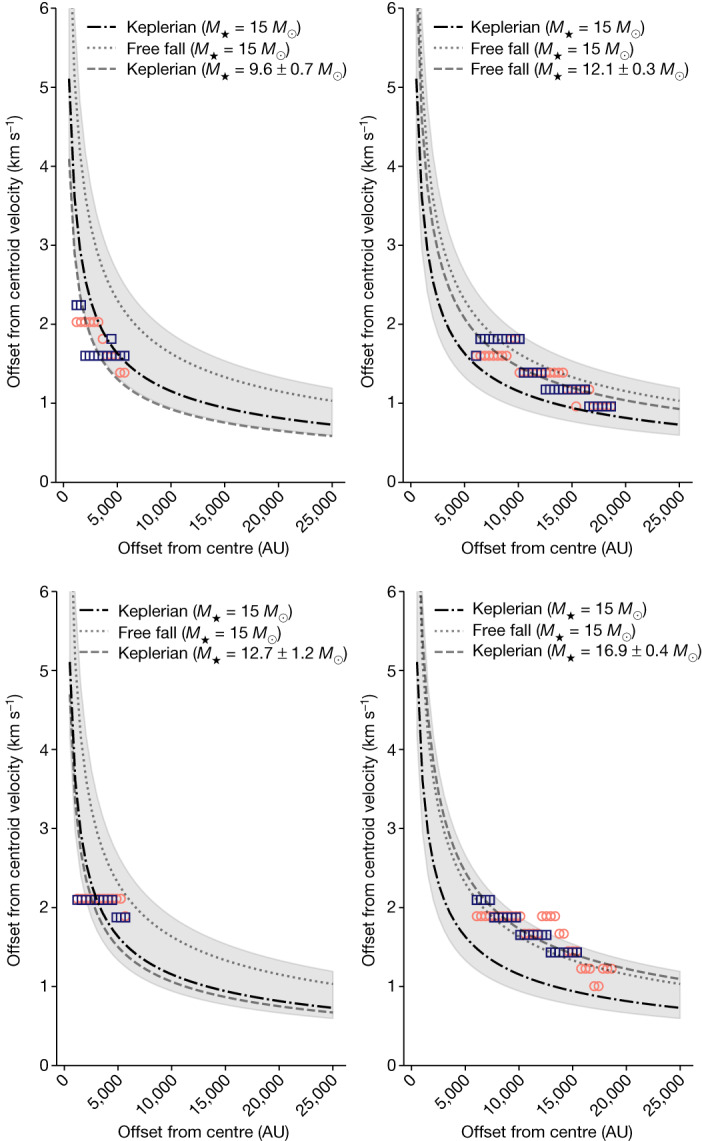
Kinematics of the inner and outer parts of the rotating structure. Kinematics in the inner regions (*R* < 6,000 AU, left panels) and outer regions (*R* > 6,000 AU, right panels) of the rotating gas. Shown are the redshifted (orange circles) and blueshifted (dark blue squares) radial velocity profiles of the outer envelope from Fig. 2c,d for CS and ^13^CO (upper and lower panels, respectively). Dot-dashed and dotted curves correspond to Keplerian and free-fall kinematics for an assumed fixed central source mass of 15 *M*_⊙_, whereas the dashed curve corresponds to the best-fitting Keplerian curves (inner regions) or free-fall curves (outer regions), showing that the inner regions are best described by Keplerian-like motion, whereas the outer regions are best described by free fall. Masses of the central source derived from the Keplerian and free-fall fits are indicated in the legend. The shaded area indicates Keplerian rotation for the mass range 10 *M*_⊙_ < *M* < 40 *M*_⊙_.

**Extended Data Fig. 3:**
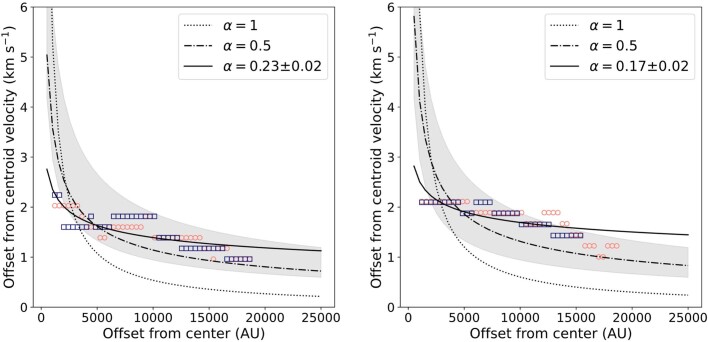
Best-fit power-laws to the gas kinematics. Best-fit power-laws to the redshifted (orange circles) and blueshifted (dark blue squares) radial velocity profiles of the outer envelope from Fig. 2. Velocities are relative to the best-fit centroid velocity of the CS (v_LSR_ = 225.3 km/s; left panel) and ^13^CO (v_LSR_ = 225.2 km/s; right panel) lines. The dotted and dot-dashed curves correspond to the best-fit power-laws with fixed index α = 1, and α = 0.5, respectively, while the solid curve corresponds to the best-fit power-law with α variable. The best-fit central source mass assuming Keplerian rotation (α = 0.5) is ~ 15 M_⊙_ and ~ 19 M_⊙_ for CS and ^13^CO, respectively. The shaded area indicates Keplerian rotation for the mass range 10 M_⊙_ < M < 40 M_⊙_.

We further explore the kinematics of the rotating gas by fitting the velocity profiles of the outer envelope with power laws^5,14^ (Methods and Extended Data Fig. 3). For both tracers, we can exclude radial infall (*v* ∝ *R*^−1^), whereas a Keplerian profile is consistent with the data, yielding an (enclosed) mass M_*_ for the central source of 14.7 ± 0.8 *M*_⊙_ and 19.5 ± 1.3 *M*_⊙_ (respectively for CS and ^13^CO). The derived enclosed stellar mass is broadly consistent with M_*_ = 12 *M*_⊙_ derived from SED fitting^13^.

Although consistent with Keplerian rotation over the extent of the rotating structure, the best-fitting power law with a variable index (as opposed to a fixed index) yields an index *α* that is considerably smaller than 0.5, indicating a combination of different kinematics (Extended Data Fig. 3). With Keplerian rotation curves describing the kinematics at smaller separations better than those at larger separations, we distinguish between an inner and an outer part of the rotating structure, using the offset of 0.12 arcsec (6,000 AU; Methods) as a critical radius. In the inner region (*R* < 6,000 AU), the kinematics are best described by Keplerian rotation (Fig. 3). This is particularly evident for CS given the better sampling of the data in this line. However, the data is insufficient to distinguish between the adopted central star mass of 15 *M*_⊙_ (obtained from the emission over the entire extent of the rotating structure) and a best-fitting Keplerian mass of approximately 9.6 *M*_⊙_ (obtained from only the emission within 6,000 AU). Large Keplerian disks with radii up to 1,000–3,000 AU have been detected around Milky Way MYSOs^2,3,6^. All except one of the most probable Keplerian disk candidates around B-type protostars (Galactic counterparts to the HH 1177 central star) also have spatially resolved disk radii in the approximately 1,000–3,600 AU regime^15^. Hence, although a factor of 2–6 larger than the aforementioned disk radii, 6,000 AU is of the same order of magnitude. In what follows, we use an outer radius of 6,000 AU as the ‘disk radius’ *R*_d_ for what we refer to as the ‘inner region’ of the rotating structure. However, we caution that the Keplerian disk is not well resolved, and the continuum source probably tracing the true disk in the inner regions, which material from the larger scales is falling onto) is only marginally resolved. We adopt *R*_d_ = 6,000 AU for further analysis as a useful limiting case.

The presence of a highly collimated bipolar jet and of rotating material transitioning to motion consistent with Keplerian dynamics around the central star of the HH 1177 system support the picture of this MYSO forming due to disk-mediated accretion and is in line with recent numerical models of a disk and jet system around massive protostars^16,17^. With all other known accreting MYSOs being in the Milky Way, the detection of the HH 1177 disk offers the opportunity to empirically analyse how the formation of massive stars might differ in an environment with comparatively lower metal and dust contents. To compare the HH 1177 system to Galactic counterparts, we computed the disk mass and accretion rate and analysed the disk stability.

**Fig. 4:**
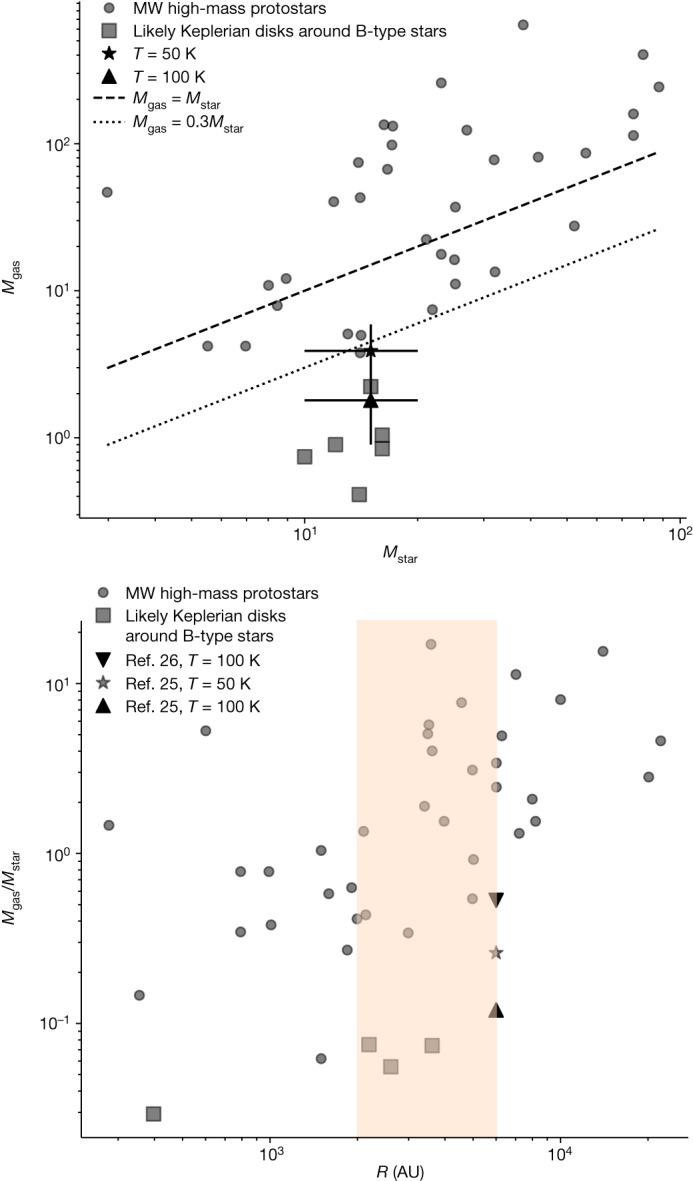
Comparison with Milky Way objects. Comparison of the HH 1177 disk to Galactic high-mass protostars (circles), with data from Beltrán and de Wit^15^. In both panels, squares correspond to the most probable Keplerian disks around (Galactic) B-type stars (all of which but one have disk radii greater than 1,000 AU) assuming Ossenkopf and Henning^25^ opacities. The upper panel shows *M*_gas_ obtained by assuming *κ*_*ν*_ from Ossenkopf and Henning and a disk temperature of 100 K (triangle) or 50 K (asterisk) together with associated errors (Methods). The lower panel illustrates different resulting *M*_gas_/*M*_star_ values for the HH 1177 source when assuming *κ*_ν_ from Ossenkopf and Henning or from Li and Draine^26^ (Methods). The asterisk and triangle are as per the upper panel. The shaded area spans disk radii of 2,000 up to 6,000 AU, that is, from radii of similar structures to the (upper limit) radius of the HH 1177 source. Not all objects indicated with squares in the upper panel are in the lower panel, as some of these do not have known stellar masses. MW, Milky Way.

We derived the disk gas mass *M*_g_ from the continuum image by assuming a gas-to-dust ratio of 380 for the LMC^18^ when converting between dust mass and gas mass. We found that *M*_g_ is between approximately 1.8 *M*_⊙_ and approximately 3.9 *M*_⊙_ by assuming a temperature of 100 or 50 K, respectively (Methods). These masses correspond to approximately 12–26% of the stellar mass. The mass of 1.8 *M*_⊙_ is consistent, within errors, with the value of approximately 0.5 *M*_⊙_ obtained from SED fitting of near-infrared observations of this source^13^. About half of the known high-mass (and intermediate-mass) protostars are above the one-to-one line in the relation between the gas mass of circumstellar structures (whether disks or toroids) and the enclosed stellar mass^15^. In this parameter space, the handful of known B-type stars with the highest likelihood of hosting Keplerian disks tend to fall below the line, having *M*_g_ < 0.3 M_*_; Fig. 4). The HH 1177 system is in the same region of the parameter space. This further supports the picture of the central star being a B-type star with a Keplerian circumstellar disk. When taking into account the disk radius (with the caveat that we are probably overestimating it), the ratio *M*_g_/M_*_ is comparable to what is found for Galactic counterparts with radii *R* > 2,000 AU (Fig. 4). This is further supported by the disk-averaged surface density (*Σ* = *M*_g_/π*R*_d_^2^) of HH 1177 and the Galactic disks with *R* > 2,000 AU being of the same order of magnitude, as the disk mass increases with *R*_d_.

**Fig. 5:**
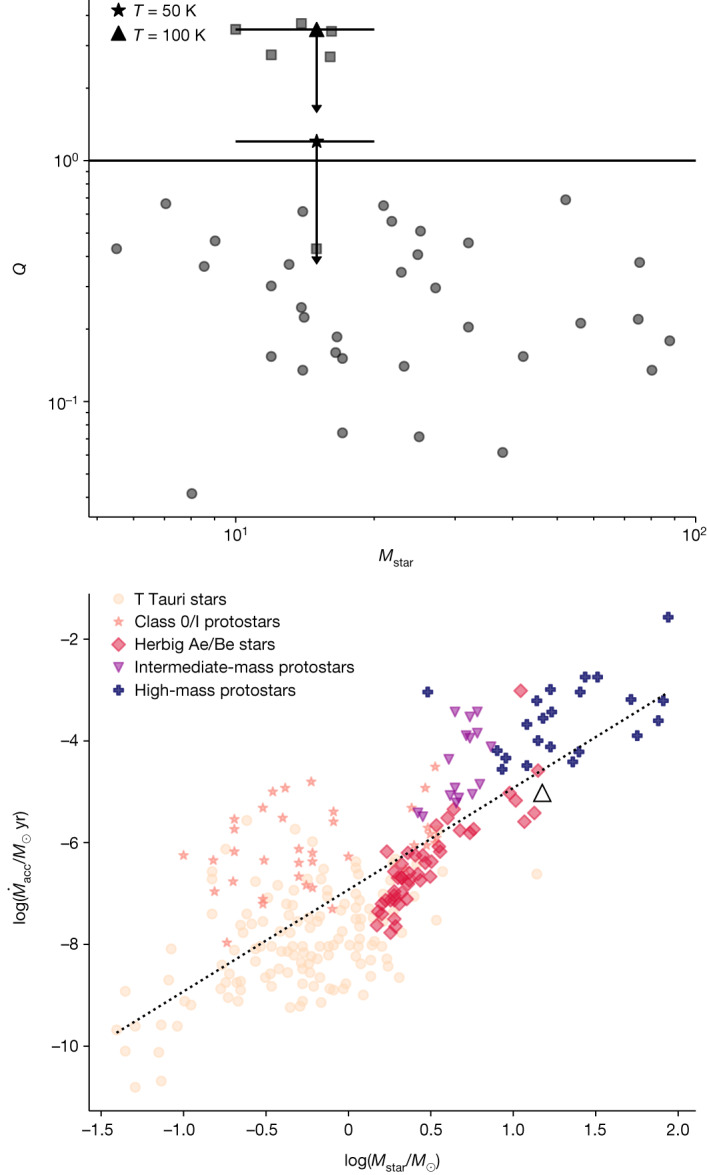
Comparison with Milky Way objects continued. Comparison of the HH 1177 disk to Galactic high-mass protostars (circles), with data from Beltrán and de Wit^15^. The upper panel shows the Toomre *Q* parameter of the HH 1177 disk as a strict upper limit, with squares corresponding to the most probable Keplerian disks around (Galactic) B-type stars assuming Ossenkopf and Henning^25^ opacities and disk temperature of 100 K (triangle) or 50 K (asterisk) together with their associated errors (Methods). The lower panel shows the relation between mass accretion rate and stellar mass for a variety of young stellar objects. The dotted line corresponds to a $\log ({\dot{M}}_{{\rm{acc}}})\propto 2\log ({M}_{{\rm{star}}})$ relation. The HH 1177 system is consistent with being an optically revealed, young, high-mass star accreting from a stable circumstellar disk.

With the disk having a mass *M*_g_ < 0.3 M_*_, the expectation of the disk being gravitationally stable^19^ is supported by a Toomre parameter *Q* > 1 (at *R*_d_ = 6,000 AU). Although this is consistent, within a factor of approximately 3, with Galactic Keplerian disks around B-type stars (Fig. 5), numerical work shows that disk fragmentation is dependent on metallicity, with strongly unstable disks^20^ resulting in higher fragmentation at lower metallicity^21^. Additionally, circumstellar disks tend to begin their lives with an unstable epoch, followed by a later epoch of stability^22^, and *Q* is a local parameter that generally decreases with disk radius. We would, therefore, expect the HH 1177 disk to have a lower Toomre *Q* parameter compared to the Milky Way B-type stars with Keplerian disks, especially at a radius of 6,000 AU. However, the opposite is the case. Although the overestimation of the disk radius and the large uncertainties stemming from the temperature assumptions play a role in producing a large value for *Q*, a possible explanation for this super-stable disk is that the HH 1177 system is exposed to the stronger radiation field at lower metallicities of the driving source compared to a Milky Way star, such that the higher photon flux might contribute to maintaining the high disk temperature and thus prevent fragmentation^23^. Although there are about 14 O-type stars in N180 (ref. ^12^), their contribution towards supporting the disk through external heating is negligible (Methods).

The growth of the central star is fuelled by accretion. For low-mass pre-main sequence stars, the mass accretion rate is proportional to the stellar mass as ${\dot{M}}_{{\rm{acc}}}\propto {M}_{* }^{1.5\mbox{--}2.0}$ (ref. ^24^). Beltrán and de Wit^15^ compared the mass accretion rates of embedded and revealed stars across a mass range spanning from low-mass pre-main sequence stars to embedded high-mass protostars. Therefore, they also sampled different evolutionary stages. They found a positive trend broadly consistent with ${\dot{M}}_{{\rm{acc}}}\propto {M}_{* }^{2}$ but also found that embedded young stellar objects have systematically higher mass accretion rates compared to pre-main sequence stars, suggesting a decrease of ${\dot{M}}_{{\rm{acc}}}$ with evolutionary stage. However, this remained inconclusive due to the lack of optically revealed high-mass stars (*M* > 10 *M*_⊙_) with accretion disks. The previously derived mass accretion rate of approximately 9.5 × 10^−6^ *M*_⊙_ yr^−1^ (derived assuming that ${\dot{M}}_{{\rm{acc}}}=3.3{\dot{M}}_{{\rm{jet}}}^{10}$) places the HH 1177 system in that previously existing gap in the parameter space (Fig. 5). It reconciles the apparent shift to higher ${\dot{M}}_{{\rm{acc}}}$ of embedded high-mass sources compared to optically revealed Herbig Ae/Be stars, therefore strengthening the probable implication that the same processes are driving accretion for all central stars and that the formation mechanism of high-mass stars is a scaled-up version of low-mass star formation.

In summary, the probable Keplerian disk of the central high-mass star driving HH 1177 is, in almost all aspects, like its Milky Way counterparts. However, it stands out for two reasons. The first is that rather than being embedded in its natal molecular cloud like all other known MYSOs, it is the only optically revealed high-mass young stellar object. The second reason is the stability of the disk. We suggest that both of these are due to the low-metallicity and low-dust content of the birth environment of the HH 1177 system, which impact the physical processes governing the optical depth of the surrounding matter. At lower metallicities, stars yield a higher number of photons in the extreme ultraviolet part of the spectrum. Owing to less efficient cooling, the temperature of the surrounding gas is higher. This results in stronger thermal pressure feedback as well as higher photo-evaporation rates. Moreover, the lower dust content decreases the overall continuum optical depth. Conversely, although we cannot exclude that the outermost radii of the disk are unstable, internal irradiation is probably responsible for maintaining a high disk temperature by stabilizing it against fragmentation. Determining the consequences of these environmental differences provides important constraints for our theoretical understanding of the formation and evolution of massive stars and their circumstellar disks.

## Methods

### Data reduction

ALMA observations for this project were taken as part of project 2019.1.00756.S. To resolve the expected Keplerian rotation, we required a spatial resolution of at least 0.05″ within a pillar that emits on 4″ scales. To capture emission on those large size scales with that resolution required two ALMA configurations. The larger-scale observations were taken on 7 and 8 October 2019, and the high-resolution data were observed on 25 and 21 October and 1 November 2021. The two sets of observations were reduced using the standard ALMA pipeline and combined in the *uv* plane using the CASA command *concatvis*. Both datasets were imaged and cleaned independently to verify their signal and noise levels. The combined dataset was successfully imaged, but the cleaning algorithms failed. They increased the noise and failed to find a signal, even though we tried several cleaning methods, such as multi-scale cleaning, using various clean boxes and manual cleaning. Thus, for this paper, we use the dirty images and note that the flux levels derived from the combined dirty images are consistent with those of the low-resolution clean images and higher than those of the high-resolution clean images, which suffer from spatial filtering. The morphologies, kinematics and mass estimated from the kinematics are not affected.

### SCOUSEPY and ACORNS decomposition

Due to the presence of several velocity components (in both ^13^CO and CS), which vary between one to three components on a pixel-by-pixel basis, we used the multi-component spectral line decomposition algorithm SCOUSEPY^27,28^ (Semi-automated multi-Component Universal Spectral-line fitting Engine) to fit the spectral line data. In brief, SCOUSEPY uses a semi-automated step-by-step approach for producing a parametric, pixel-wise, multi-component description of spectroscopic data cubes. We performed a Gaussian decomposition of the ^13^CO and CS data cubes. To define the coverage for the SCOUSEPY decomposition, we masked the data cubes at a level of 0.007 or 0.0035 Jy per beam. We generated spectral averaging areas (SAAs) of size 20 and 10 pixels, resulting in 356 and 207 SAAs, respectively, with the larger size of the ^13^CO SAAs reflecting the more extended emission. Of these SAAs, a total of 25 and 41 were decomposed manually, whereas the remaining SAAs made use of SCOUSEPY’s derivative spectroscopy methodology (Henshaw et al., in preparation). Of the 34,660 and 5,600 spectra contained within the ^13^CO and CS data cube coverage, 34,560 and 5,314 have model solutions. Of these model solutions, the fractional number of pixels requiring multi-component models is small (of the order of 10%). However, the maximum number of components identified within a single spectrum can be as high as four, justifying the need for Gaussian decomposition.

Given the presence of several velocity components in our data, we next used ACORNS (Agglomerative Clustering for Organising Nested Structure) to cluster the extracted components into velocity-coherent features. ACORNS is based on a technique known as hierarchical agglomerative clustering, which generates a hierarchical system of clusters of data connected in *n*-dimensional space using selected properties. For our parametric description of the velocity components output from SCOUSEPY, we clustered these data based on the separation between data points in physical space, their velocity centroids and their velocity dispersion. We set the linking lengths for clustering based on the observational limitations of our data. Specifically, we defined the minimum cluster size to 25 pixels and set the linking length in velocity and velocity dispersion to be twice the spectral resolution of our data. From the resulting hierarchy, we extracted the largest clusters identified by ACORNS, which are displayed in Fig. 2a,b. Although most of the pixels display a single component (more than 92% for ^13^CO and more than 88% for CS), all pixels are shown in Fig. 2a,b.

### Outer envelope analysis, mass of central source and gas kinematics

The mass of the star was estimated according to the method described in Seifried et al.^14^, under the assumption that the motion of the disk follows Keplerian rotation. Briefly, this method consists of first estimating the mean level of the noise *σ*_rms_ in the outer parts of the PV diagrams (where there is no emission). Based on this, for each radial offset position in the PV diagram starting from the offset position at the highest velocity (or the lowest, for the opposite quadrant), it identifies the first pixel with emission above the adopted threshold. The velocity of this pixel corresponds to the maximum (minimum) rotation velocity at that offset radius. This results in the identification of the outer edge of the rotating structure in the form of the maximum rotation velocity as a function of positional offset. This can then be used to constrain the mass of the central source based on the best-fitting (Keplerian) curve to the extracted data points.

**Extended Data Fig. 4:**
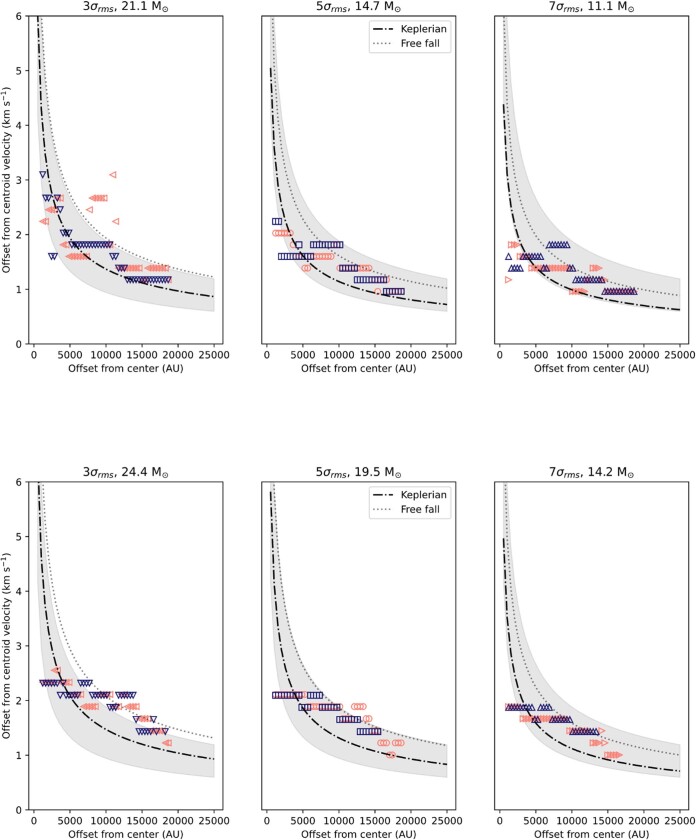
Rotation curves at different density thresholds. Keplerian (dot-dashed) and free fall (dotted) curves for sources with central masses derived from the best fit α = 0.5 power law to the redshifted (orange) and blueshifted (dark blue) radial velocity profiles of the outer emission envelope from Fig. 3 (CS and ^13^CO in the upper and lower panels, respectively). The three panels correspond to different background noise thresholds used in determining the maximum rotation velocity^14^ with the left showing a 3σ_rms_, the central panel the adopted 5σ_rms_, and the right a 7σ_rms_ threshold. Velocities are relative to the best-fit centroid velocity of the CS (v_LSR_ = 225.3 km/s) and ^13^CO (v_LSR_ = 225.2 km/s) lines. The shaded area indicates Keplerian rotation for the mass range 10 M_⊙_ < M < 40 M_⊙_.

We found mean levels of noise *σ*_rms_ of 1.5 and 0.4 mJy for ^13^CO and CS, respectively. We adopted a threshold of 5*σ*_rms_, which produces the ‘cleanest’ velocity versus offset data (middle panels in Extended Data Fig. 4). Figure 2c,d shows the PV diagrams for the two species and the resulting outer edge of the structure, together with curves expected for Keplerian motion (teal lines) and free-fall motion (pink lines) around a central source of different masses.

The redshifted and blueshifted sides of the outer envelope are folded into a single plot in Extended Data Fig. 3 to allow further analysis of the kinematics of the structure. We fitted a power law to the PV diagram of the form *v* = *βR*^*−α*^, where *α* = 0.5 corresponds to Keplerian and *α* = 1 to infall motion. Additionally, we performed a fit in which the exponent α is allowed to vary. Although the data are clearly not described by infall, the fits demonstrate that the kinematics is probably a combination of Keplerian rotation (or sub-Keplerian, with *α* ≈ 0.2) and free fall. The best-fitting central source mass for Keplerian rotation is (19.5 ± 1.3) and (14.7 ± 0.8) *M*_⊙_ for ^13^CO and CS, respectively. In the following analyses, we assume a central star mass of 15 *M*_⊙_ for simplicity. We note, however, that the derived stellar mass is a lower limit due to the effect of the inclination angle *i* on the mass estimate, such that lower masses are derived from the outer envelope calculation in systems that are increasingly deviating from an edge-on viewing angle^14^. With an inclination of about 73° (ref. ^13^), the HH 1177 system is close to edge-on. Thus, we expect the derived mass to be smaller by a factor of about 0.9 (given that the fitted mass scales as cos^2^(90° − *i*)).

Further, as can be seen in Fig. 2 and Extended Data Fig. 3, the resulting outer envelope is more consistent with Keplerian motion at smaller offsets (less than about 0.15 arcsec, which is in the vicinity of the continuum source), and with free-fall motion at larger offsets. To further illustrate this, we separated the outer envelope data points into two samples describing the regions closer (*R* *<* *R*_crit_) and further away (*R* *>* *R*_crit_) from the centre. We adopted a critical radius *R*_crit_ = 6,000 AU, which is twice the mean angular resolution, and performed two fits. First, we assumed a central source mass of 15 *M*_⊙_ and fitted a Keplerian and a free-fall model to the two regimes. Second, we let the central source mass vary and fitted a Keplerian model to the inner region and a free-fall model to the outer region. This is summarized in Fig. 3. For CS, although we cannot rule out free-fall motion with a central mass of less than about *M*_⊙_, the inner regions are clearly better described by Keplerian rotation with a central star in roughly the mass regime expected from the literature. The opposite is true for the outer regions traced by CS. Although we cannot rule out Keplerian rotation with a central mass greater than about 2 *M*_⊙_, free-fall motion with a central source of the expected mass is a good description. Although less predictive, the same argument is valid for ^13^CO. We, therefore, suggest that the gas in the detected rotating structure is free-falling from the outer regions onto a central disk where the kinematics are (sub-)Keplerian. The scale of a few thousand astronomical units at which the transition to a Keplerian disk occurs are consistent with the size of Keplerian accretion disks observed around massive stars^15^.

### Masses of the inner disk and outer envelope

The area used for to derive a source flux for the mass calculation below was derived using the CASA task ‘imfit’. This task fits a two-dimensional Gaussian to the emission in the continuum image and reports the area (deconvolved from the synthesized beam) and enclosed flux. We have

$${M}_{{\rm{dust}}}=\frac{{d}^{2}{F}_{\nu }}{{\kappa }_{\nu }{B}_{\nu }\left({T}_{{\rm{dust}}}\right)}$$

where *d* is the distance to the source, *F*_*ν*_ is the integrated flux of 0.34 mJy of the continuum source (as described above), *κ*_*ν*_ the dust opacity per unit mass at a frequency *ν*, and *B*_*ν*_ the Planck function at a temperature *T*_dust_.

The main sources of uncertainty in the estimation of the dust mass stem from *κ*_*ν*_ and *T*_dust_ (ref. ^15^). Here we used a dust opacity from Ossenkopf and Henning^25^ (see below), which differs from Johnston et al.^2^, who used opacities from Draine^29^ (which are systematically lower than the Ossenkopf and Henning values and probably more suitable for diffuse clouds^6^), but is in line with other studies of disks around massive stars^3,6,30^ and consistent with the Beltrán and de Wit review^15^ for comparative purposes. All opacities assume Galactic metallicity.

We computed the disk mass and subsequent parameters assuming two temperatures. First, we assumed a temperature of 100 K, based on the properties of other probable Keplerian disks of similar central stars and sizes^31,32^. Second, we derived a disk temperature from radiative equilibrium (equating cooling and heating rates^33^):

$${T}_{{\rm{dust}}}={\sigma }^{-1/4}{(1-\alpha )}^{1/4}{\left(\frac{{L}_{{\rm{star}}}}{4\pi {R}^{2}}\right)}^{1/4},$$

where *α* is the albedo (assumed to be 0.6), *σ* is the Stefan–Boltzmann constant, and *L*_star_ and *R* are the luminosity of and the distance from the central star, respectively. Based on the central source parameters (temperature and radius) derived from SED fitting^13^, we assumed *L*_star_ ≈ 1.9 × 10^4^ *L*_⊙_ based on the best comparable LMC stellar atmosphere model (model 28–40 in Hainich et al.^34^). This luminosity is comparable to that quoted for Galactic B-type stars bearing Keplerian disks^15^. Inserting *R* = 6,000 AU yields *T*_dust_ ≈ 50 K. Although there are about four O-type stars within a (projected) radius of 12 pc, their contribution to heating the disk is negligible (their contribution to the equation above would be in the form of an added *L*_star_/4π*D*^*2*^ term, with *D* being the projected distance to the external sources). We note that the disk temperature is radius-dependent, such that assuming a single temperature leads to large uncertainties in both the disk mass and the stability calculation, as described next.

We adopted a distance of 49.59 kpc to the LMC^35^ and a dust opacity at 0.87 mm of approximately 2.5 cm^2^ g^−1^ (assuming grains with thin ice mantles and coagulation at 10^8^ cm^−3^). The dust mass was then converted into a gas mass as *M*_gas_ = GDR × *M*_dust_, where GDR is the gas-to-dust ratio, which for the LMC is ${\rm{GDR}}={380}_{-130}^{+250}$ (ref. ^18^). To compute the uncertainty for the derived gas mass, we assumed a 20% error for the flux measurement and propagated these errors together with the GDR uncertainties. The derived disk gas mass is, thus, 1.8 ± 0.9 *M*_⊙_ at 100 K and 3.9 ± 2.0 *M*_⊙_ at 50 K. These correspond to approximately 12% and 26% of the stellar mass (assuming *M*_star_ ≈ 15 *M*_⊙_; see above). Although the disk gas mass under the 50 K assumption is on the high end compared to similar Galactic sources, the computed *M*_gas_ for 100 K is consistent, within errors, with the value of approximately 0.5 *M*_⊙_ obtained from SED fitting^13^. However, the corresponding disk radius of about 100 AU obtained from the SED modelling is significantly smaller than the rotating structure observed here, supporting the likelihood of a radius of 6,000 AU being a strict upper limit.

### Toomre stability analysis

We assessed the stability of the structure consistent with Keplerian rotation (*R* ≲ 6,000 AU, that is, the disk) using the Toomre *Q* instability parameter^36^, which for Keplerian rotation is defined as

$$Q=\frac{{c}_{{\rm{s}}}\varOmega }{\pi G\varSigma }$$

where *c*_s_ is the speed of sound, $\varOmega =\sqrt{G{M}_{{\rm{tot}}}/{R}^{3}}$ is the angular velocity of the disk (with *M*_tot_ the combined mass of the central star and the disk) and *Σ* the surface density, which is calculated as *Σ* = *M*_disk_/π*R*^2^. For temperatures of 100 K and 50 K (see above), a radius of 6,000 AU, and adopting the derived disk and stellar masses, we obtain *Q* = 3.5 ± 1.8 or *Q* = 1.2 ± 0.8 (for 100 and 50 K, respectively). Although both values are consistent with disk stability (*Q* > 1), we cannot exclude that the outermost disk radii are unstable, due to the large uncertainties stemming from both the adopted disk radius (which is a strict upper limit) and the assumed central star luminosity. However, given that the disk temperature is radius-dependent, as described above, the smaller radii become increasingly stable. If the radius of the disk is less than 6,000 AU, the disk temperature at the outer radii increases and the disk probably becomes stable at all radii. Detailed modelling of disks around massive stars at low metallicities will be necessary to quantitatively understand these types of systems.

## Online content

Any methods, additional references, Nature Portfolio reporting summaries, source data, extended data, supplementary information, acknowledgements, peer review information; details of author contributions and competing interests; and statements of data and code availability are available at 10.1038/s41586-023-06790-2.

## Data Availability

The authors declare that the data supporting the findings of this study are publicly available from the ALMA data archive under the project 2019.1.00756.S.

## Extended data

Extended data are available for this paper at 10.1038/s41586-023-06790-2.

## Code availability

All code generated for this study is available from the corresponding author upon request.
